# Fat Pad Entrapment at the Hip: A New Diagnosis

**DOI:** 10.1371/journal.pone.0083503

**Published:** 2014-02-26

**Authors:** Narlaka Jayasekera, Alessandro Aprato, Richard N. Villar

**Affiliations:** Villar Bajwa Practice, Spire Cambridge Lea Hospital, Cambridge, United Kingdom; University of Leicester, United Kingdom

## Abstract

**Purpose:**

To establish if a positive impingement sign in femoroacetabular impingement (FAI) may result from entrapment of the fat pad located at the anterior head-neck junction of the upper femur. This fat pad is routinely removed before any cam lesion excision.

**Methods:**

We report a prospective study of 142 consecutive hip arthroscopies for symptomatic FAI where the aim was to remove the arthroscopically identified area of impingement, not necessarily to create a spherical femoral head. Patients were divided into two groups. Group 1 (n = 92; 34 females, 58 males), where a cam-type bony FAI lesion was identified and excised in addition to the fat pad which overlay it, and Group 2 (n = 50; 29 females, 21 males) where the only identified point of impingement was a prominent fat pad. In this situation the fat pad was excised in isolation and the underlying bone preserved. Patients were assessed preoperatively, at six weeks, six months, one year and two years with a modified Harris hip score (mHHS).

**Results:**

Both groups were comparable preoperatively for mean age, mean alpha angle and mean anterior offset ratio. Both groups improved significantly after surgery at all time points. However, Group 1 (fat pad and bone resection) demonstrated 16.0% improvement in mHHS by two years while for Group 2 (fat pad resection only) the improvement was 18.9% (p = 0.628).

**Conclusions:**

The fat pad found at the anterior head/neck junction of the hip joint can be a source of pain and we propose fat pad entrapment as a new, previously undescribed diagnosis. Our findings also suggest that a large number of cam lesions are being excised unnecessarily and that further efforts should be made to understand the role of the fat pad as a source of groin discomfort.

**Level of Evidence:**

Level IV, case series.

## Introduction

Recent expansion in hip arthroscopy has been driven by a claimed association between labral tears, femoroacetabular impingement (FAI) and early-onset osteoarthritis (OA) [1]–[9]. When assessing a patient with FAI it is common practice to undertake the so-called impingement test, performed by passive hip flexion, adduction and internal rotation with the patient supine [10]. Groin pain created by this is thought to arise from a torn labrum [11] or an area of chondral injury coming into contact with the proximal and anterior part of the femoral neck [11], or a bony cam deformity [12]. However, anatomically a fat pad may be found at the anterior head-neck junction of the upper femur. This fat pad is routinely removed at surgery before any cam lesion is excised. Meanwhile, fat pad entrapment has been well described in the knee [13] where acute and repetitive trauma to the infrapatellar fat pad first causes haemorrhage [13], [14]. The inflamed fat pad then becomes hypertrophied, thus predisposing it to further entrapment within the joint [13].

Our hypothesis was that some patients with a positive impingement sign are not experiencing pain from their cam deformity or labral tear at all, but from fat pad entrapment. This concept has not been previously described. We also aimed to investigate the short-term outcome after arthroscopic excision of a cam–type FAI lesion and to compare it with arthroscopic debridement of the fat pad at the femoral head-neck junction but without the excision of a bony cam-type FAI lesion.

## Methods

**Figure 1:**
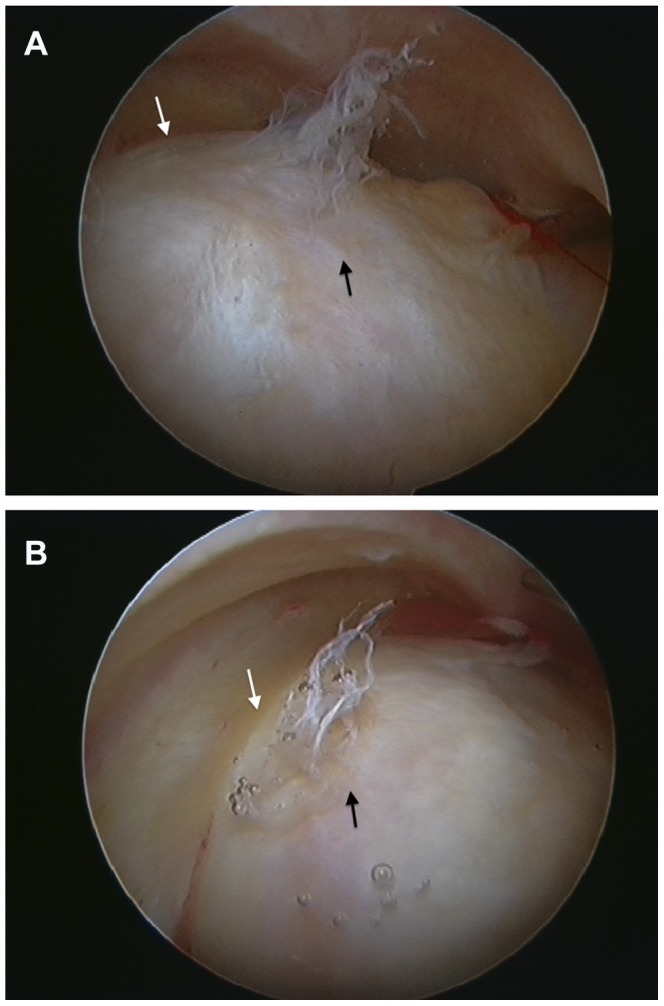
A cam-type impingement lesion (black arrow) and adjacent labrum (white arrow) of the right hip viewed from the peripheral compartment during hip arthroscopy. Figure 1A. Femoral head-neck junction. Figure 1B. Femoroacetabular impingement demonstrated with the hip flexed to 90°. Note deformation of the labrum.

**Figure 2:**
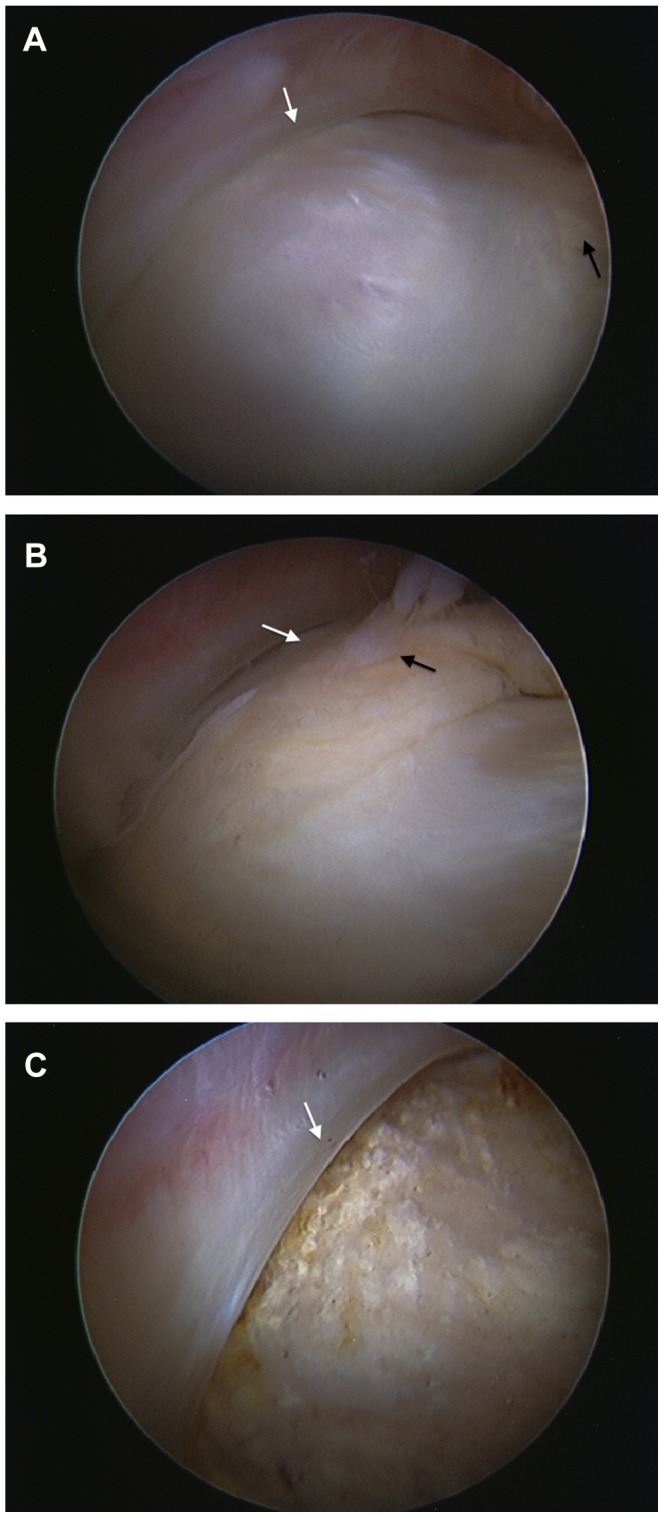
The labrum (white arrow) and prominent fat pad (black arrow) of a right hip. The anterolateral femoral head/neck junction as viewed from the peripheral compartment. Figure 2A. View of femoral head-neck junction and labrum. Figure 2B. Entrapment of the prominent fat pad with the hip in 90° flexion. Figure 2C. Excision of the fat pad almost complete and impingement between labrum and head-neck junction now relieved.

We investigated 142 consecutive patients who underwent arthroscopy of the hip for symptomatic FAI in the specialist practice of the senior author. All patients had a positive impingement test elicited by the senior author, and had failed to respond symptomatically to conservative treatment, which included activity modification, physiotherapy and treatment with nonsteroidal anti-inflammatory agents. A positive impingement test is defined as the finding of sharp pain in the ipsilateral groin on passive adduction and internal rotation of the hip held in at least 90° flexion [10]. All patients underwent standard anteroposterior (AP) pelvic and lateral hip radiographs and either MRI or MR arthrography within six months of their first outpatient visit. A cam-type impingement lesion was defined as abnormal asphericity or reduced offset of the anterolateral femoral head-neck junction, measured from the MRI scan using a digital goniometer and ruler before surgery to provide the alpha angle [15] and anterior offset ratio, [16], [17] respectively. Operations were performed under general anaesthetic in the lateral position with a specialist hip distractor (Lateral Hip Positioning System, Smith & Nephew Inc., Andover, Massachusetts, USA) [18]. Routine dynamic peroperative arthroscopic assessment was performed by repositioning the hip to 90 degrees of flexion. Impingement was deemed to be present if the labrum was seen to lift as the femoral head passed to and fro beneath it. By this means it was possible to identify arthroscopically the anatomical point at which impingement was occurring. On this basis, patients could be subdivided into two groups. Group 1, where a bony cam-type FAI lesion was seen to be the cause of impingement (Figures 1A and 1B), irrespective of whether or not a fat pad overlay it. For these patients, both the fat pad and the underlying bony lesion were excised with a combination of radiofrequency and a 5.5 mm high-speed spherical burr (Dyonics High Visibility Sheath Abrader Burr, Smith & Nephew Inc, Andover, Massachussets, USA). Group 2, however, comprised those patients where the only source of impingement was a prominent fat pad at the anterior femoral head/neck junction but where there was no evidence of a bony lesion impinging at all (Figures 2A, 2B and 2C). Even if a bony lesion was present, it could not be seen to form part of the impingement process during dynamic peroperative assessment under direct arthroscopic view. For these patients, only the fat pad was excised with a 90° radiofrequency tissue ablator (Dyonics RF-S Whirlwind, Smith & Nephew Inc, Andover, Massachussets, USA) but the underlying bone was preserved. For both groups, excision was considered complete when the femoral head/neck junction was seen to pass smoothly beneath the labrum on dynamic peroperative assessment without any points of impingement being seen. The aim of this procedure was to remove the area of impingement, not necessarily to create a spherical femoral head.

All patients underwent counseling and assessment by a physiotherapist prior to surgery. All operative findings were assessed and described by the senior author and recorded in a custom-made database (Microsoft 2010). All patients gave written consent that their data could be stored in this way and that these data could be used for future studies. All lesions were photographed and any associated pathology within the joint was also recorded. Both groups underwent an identical physiotherapy regimen postoperatively, which allowed patients to partial weight bear with crutches on the very day of surgery under supervision of the physiotherapist. Crutch use was continued for four weeks thereafter and postoperative physiotherapy continued for at least 12 weeks after surgery. For the purpose of this study partial weight was defined as the use of crutch support when weight bearing on the operated hip. Data were collected prospectively and the outcome measure used was the modified Harris hip score (mHHS). This was recorded pre-operatively, at six weeks, six months, one year and two years after surgery. The mHHS has construct validity for hip arthroscopy [19]. Also, it is the most frequently used outcome score in hip arthroscopy indicated by intra-articular pathology [20].

All patients’ data were analysed by a commercial software package (TexaSoft, WINKS SDA Software, 6th Edition, Cedar Hill, Texas, 2007). Normal data distribution was checked by the test for equality of variance. Independent samples t-test was used for the analysis of age, alpha angle, anterior offset ratio and mHHS between groups. Statistical significance was fixed at p<0.05 for all tests performed.

### Ethics statement

Spire healthcare review board clearance was obtained for this study and consent process. All patients provided written informed consent, which was retained in their clinical records.

## Results

In Group 1 (fat pad and bone resection; 34 females, 58 males) the mean age was 39.5 years (18 to 58), the mean pre-operative alpha angle was 60° (35° to 71°), mean pre-operative anterior offset ratio was 0.31 (0.11 to 0.50) and the mean pre-operative mHHS was 62 (28 to 87). In Group 2 (fat pad resection only; 29 females, 21 males) the mean age was 39 years (16 to 68), the mean pre-operative alpha angle was 59° (33° to 90°), mean pre-operative anterior offset ratio was 0.35 (0.10 to 0.73) and the mean pre-operative mHHS was 53 (31 to 87). A summary of the pre-operative diagnoses and intra-operative interventions is shown in Tables 1 and 2, respectively.

**Table 1:** Summary of preoperative diagnoses.

Pathology	Group 1	Group 2
Femoroacetabular impingement	92	50
Chondral lesion	39	22
Labral tear/abnormality	53	18
Osteochondral defect	9	4
Iliopsoas tendinopathy	5	2
Loose/foreign bodies	3	1
Synovitis	10	5
Partial tear of ligamentum teres	3	2
Periarticular cyst	2	3
Snapping iliotibial band	3	2

**Table 2:** Summary of operative interventions.

Procedure	Group 1	Group 2
Excision of cam-type lesion	92	0
Excision of fat pad	92	50
Acetabular recession	9	2
Microfracture	18	2
Chondroplasty/chondral repair	67	31
Partial labrectomy	56	19
Repair/reattachment of labrum	13	4
Decompression/lengthening of Iliopsoas	9	5
Removal of loose bodies	5	2
Synovectomy	13	6
Shrinkage/debridement of ligamentum teres	6	3
Decompression of periarticular cysts	5	4
Iliotibial band release	3	2

There was no significant difference between the groups in mean age (p = 0.862), mean pre-operative alpha angle (p = 0.723) or mean pre-operative anterior offset ratio (p = 0.392). However, there was a significant difference in the mean pre-operative mHHS between the two groups with Group 1 being less incapacitated pre-operatively than Group 2 (62 points *vs* 53 points, respectively; p = 0.01). For Group 2, there was a greater improvement in the mean post-operative total mHHS at six weeks, six months and two years (Table 3), although only the six-week score was significantly better than Group 1 (p = 0.014, p = 0.56, p = 0.63, respectively. Group 1 patients demonstrated a 16.0% improvement in their mHHS from their preoperative level by two years after surgery while for Group 2 their improvement was 18.9%. This difference was not significant (p = 0.63).

**Table 3:** Improvement in mean modified Harris Hip Scores for Groups 1 and 2.

Time from surgery	Mean mHHS Group 1	Mean mHHS Group 2	p value
6 weeks	2 (−6 to 45)	13 (−3 to 51)	0.014
6 months	8 (−4 to 43)	14 (−2 to 47)	0.56
1 year	12 (−1 to 20)	9 (0 to 13)	0.18
2 years	13 (0–23)	25 (3 to 54)	0.63

Range in improvement of mHHS is shown in brackets, negative scores denote a drop in mHHS. mHHS; Modified Harris Hip Score.

## Discussion

Our results suggest that the fat pad of the hip can be a source of pain and that in properly selected cases, excision of the fat pad alone within the peripheral compartment is sufficient to improve a patient’s symptoms. We have also shown that to solely remove the point of impingement is sufficient and there may be no need to refashion every femoral head to perfect sphericity.

Our Group 2 patients, those in whom only the fat pad was removed, had significantly better improvement in their mHHS at the six-week point after surgery when compared with the Group 1 patients, where an osteochondroplasty had been performed. One might expect this, as the removal of a fat pad requires much less surgical invasion than the removal of a fat pad plus any underlying bony bump. By the six-month point a continued improvement in the mHHS of both groups was noted. A similar pattern of improvement in mHHS was observed in both groups for the subsequent duration of this study.

There is now an increasing appreciation of the wider spectrum of normal hip morphology [21]. Pollard et al [17], in a general mixed-gender population, derived a 95% reference interval for the normal alpha angle of 32° to 62°. The normal range for the anterior offset ratio was 0.18 to 0.24. The mean alpha angle for both groups in our study fell within the reference range, thus demonstrating the dilemma experienced by the clinician who chooses to rely on radiographic measurements in the management of FAI. Conversely, the mean anterior offset ratio for both groups in our study was above the normal reference range.

Although the fat pad at the anterior femoral head/neck junction is a familiar structure to the hip arthroscopist, to-date it remains undescribed in the surgical anatomical literature [22]. This is unlike its counterpart in the knee, which has been described in detail over the last century [13], [14], [23]–[25] and shows growing evidence of a role in the pathophysiology of degenerative joint disease [26]–[28]. The fat pad at the anterior femoral head/neck junction may have suffered neglect owing to its small size and impression of relative inconsequence. However, as with the infrapatellar fat pad in the knee, the fat pad of the hip may become repeatedly traumatised by the labrum and acetabular margin during the impingement process. This may be of considerable significance in the pathogenesis of intra-articular hip pathology. Certainly the fat pad can be a source of pain, a feature clearly highlighted by our own study.

The infrapatellar fat pad, or Hoffa’s fat pad, is an intracapsular, extrasynovial structure within the anterior compartment of the knee. It is highly vascularised and innervated and may be capable of modulating inflammatory and destructive responses in OA of the knee [26], [28]. Our suggestion is that the fat pad of the hip may play the same role as the fat pad of the knee in the establishment of OA.

There are a number of limitations to this study. It is of single centre origin and from a single experienced hip arthroscopic surgeon using an identical technique and rehabilitation protocol. This may influence the reproducibility of results [29], as a less experienced surgeon may face a learning curve for appropriate patient selection and the procedure itself. Although data were collected prospectively in our study, neither patient nor assessor was blinded to the surgical intervention with risk of bias in the postoperative mHHS and postoperative clinical assessment. Our study did not include a group that received hip arthroscopy but no treatment for bony impingement or fat pad entrapment. Follow-up in our study was limited to two years. Further studies of longer follow up are necessary to establish the longer-term clinical outcomes.

## Conclusions

It thus appears that the fat pad found at the anterior head/neck junction of the hip joint can be a source of pain and we propose fat pad entrapment as a new, previously undescribed diagnosis. Our findings also suggest that a large number of cam lesions are being excised unnecessarily and that further efforts should be made to understand the role of the fat pad as a source of groin discomfort.
